# Coupling fragmentation to a size-selective sedimentation model can quantify the long-term fate of buoyant plastics in the ocean

**DOI:** 10.1098/rsta.2024.0445

**Published:** 2025-10-23

**Authors:** Nan Wu, Stuart Grieve, Andrew Manning, Kate Spencer

**Affiliations:** ^1^ School of Geography, Queen Mary University of London, London, UK; ^2^ British Antarctic Survey, Cambridge, UK; ^3^ Digital Environment Research Institute, Queen Mary University of London, London, UK; ^4^ HR Wallingford Ltd, Wallingford, UK; ^5^ School of Biological and Marine Sciences, University of Plymouth, Plymouth, UK

**Keywords:** plastic degradation, microplastic sedimentation, marine snow aggregates

## Abstract

Plastic pollution in the ocean is a global environmental issue, with buoyant debris accumulating at the surface and posing long-term ecological threats. Although sediments are the ultimate sink for plastics, a mismatch between observed surface concentrations and estimated inputs implies the understanding of vertical sedimentation mechanisms and rates are inaccurate. Here, we present a coupled fragmentation–sedimentation model that quantitatively predicts the vertical transport and long-term fate of buoyant plastic debris and microplastics (MPs, less than 5 mm). Using a representative 10 mm polyethylene (PE) particle, we show that fragmentation into small MPs is essential for their incorporation into marine snow aggregates (MSAs) and subsequent settling. Even after 100 yr, ca. 10% of the initial plastic mass still remains at the surface providing a continual source of small MPs to ocean surface waters. This study provides the first mechanistic framework linking large plastic degradation to size selective sedimentation, demonstrating that plastic pollution will persist at our ocean surfaces for over a century even if inputs cease. Our findings highlight the need for mitigation strategies beyond input reduction and ocean clean-up, addressing the long-term removal of existing ocean plastics.

This article is part of the Theo Murphy meeting issue ‘Sedimentology of plastics: state of the art and future directions’.

## 1. Introduction

Large buoyant plastics (greater than 5 mm) dominate the ocean surface, and are collectively estimated at approximately 250 000 metric tonnes globally [1]. Due to their high buoyancy and resistance to vertical sinking processes, these plastics can persist at the ocean surface for decades [2–5]. Their prolonged residence at the ocean surface exposes them to continuous degradation via photo-oxidation, mechanical fragmentation and microbial degradation, resulting in the gradual release of small microplastics (MPs, less than 5 mm) into the ambient environment [6–8]. These released MPs pose an ecological risk across marine trophic levels and can potentially interfere with ocean biogeochemical cycles [9–11]. Despite recognizing these significant effects, we lack a detailed understanding of the rates and mechanisms by which buoyant plastics (and their derived MPs) are transported to deep ocean sediments and there is a mismatch between estimated ocean input of plastics and concentrations observed at the surface.

The plastic component of deep marine sediments is dominated by small, buoyant MPs (less than 100 µm) [12–15], and the distribution of MPs in the water column is strongly associated with particulate matter, especially marine snow aggregates (MSAs) [12,16,17]. MSAs (organic-rich particles) play a critical role in the gravitational settling of materials from the ocean surface to the deep sea [18,19]. Therefore, this suggests that fragmentation of plastic litter, followed by incorporation into MSAs is a critical pathway for the transportation of buoyant plastics to the deep ocean [20–23].

We have established a predictive model based on an extensive meta-analysis of data (more than 2000 measurements) and experimental validation (more than 4000 measurements) that demonstrates the incorporation of MPs into sedimentary flocs or aggregates is dependent on a simple relationship between MP size and floc size, and that this relationship holds for wide-ranging aquatic environments [24]. This model revealed that MPs smaller than approximately 160 µm are preferentially incorporated into MSAs, and is defined mathematically in equation (1.1).

For aggregate size (*x*, µm), the threshold MP size (*y*, µm) is:


(1.1) $$\begin{array}{rl} 𝑥\leq 900,𝑦= & \;-0.0002{𝑥}^{2}+0.36𝑥,\\ x>900,\;y= & \;162. \end{array}$$

We have also developed a detailed mechanistic sedimentation model that demonstrates how small, buoyant MPs can only ultimately accumulate in deep ocean sediments through repeated aggregation–disaggregation cycles with MSAs [23].

We hypothesize that large buoyant plastics at the ocean surface degrade until they reach a critical threshold of approximately 160 µm and are subsequently size-selected and transported into deeper ocean layers through marine snow sedimentation. We have coupled these two new advances in understanding the mechanistic transport of MP [23,24] with an advanced degradation model [25] to predict the long-term fate of buoyant plastic waste at the ocean surface.

Specifically, the objectives of this study are: (i) model the degradation and fragmentation processes of large buoyant plastics at the ocean surface, predicting resultant MP size distributions; (ii) simulate the vertical sedimentation and redistribution of these degraded, buoyant MPs within the ocean interior, quantifying their distribution across ocean depths; (iii) estimate the longevity and surface retention time scales of large plastic debris, providing essential data for understanding the natural removal processes from the ocean surface. This integrated approach significantly enhances our ability to forecast plastic debris behaviour in marine environments, ultimately informing strategies for managing ocean plastic pollution.

## 2. Methods

**Figure 1.**
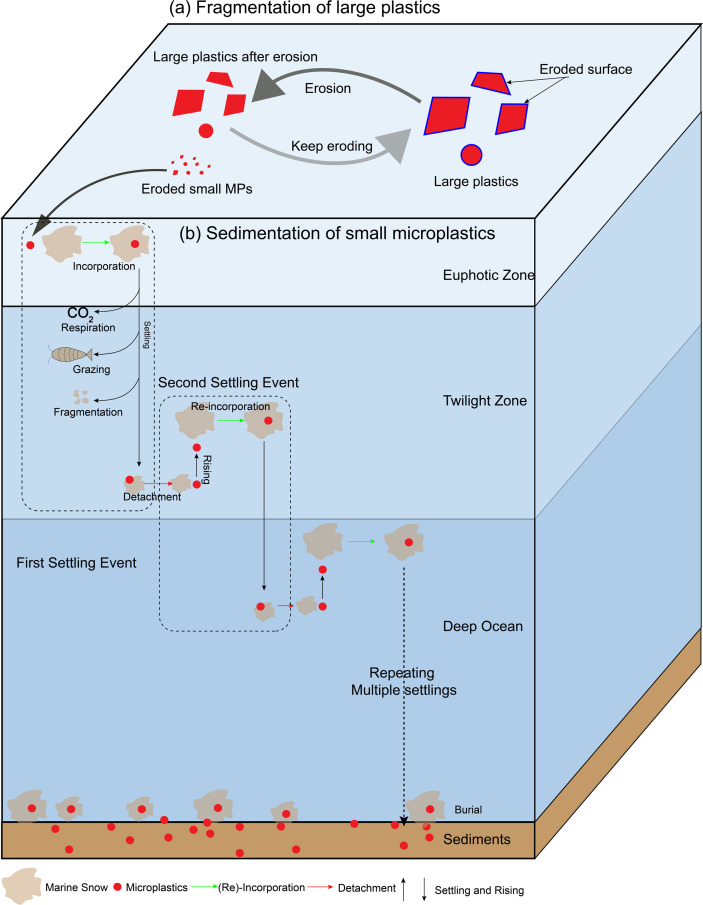
Schematic diagram of how large plastic waste reaches deep-sea sediments by combining these components: (a) the degradation and fragmentation of large plastic waste at the ocean surface and the fragmented MPs can be incorporated into marine snow and taken to deep sea based on equation (1.1) as (b) the settling of fragmented MPs by MSAs in the ocean interior [23]. The MPs will stay at the ocean surface for further fragmentation (a) if they are not able to be incorporated. The layers of water column: Euphotic zone: 0−100 m. Upper twilight zone: 100−500 m. Lower twilight zone: 500−1000 m. Mid-layer: 1000–2000 m. Deep sea: 2000−5100 m.

The one-dimensional model was developed to simulate the long-term degradation and sedimentation of buoyant plastic waste from the ocean surface, explicitly linking surface fragmentation to biologically mediated vertical transport. The model operates along a 5100 m deep water column, representative of the North Pacific Subtropical Gyre, where floating plastic debris accumulates. It tracks the evolution of one polyethylene (PE) microsphere (diameter = 10  mm), modelling how it gradually breaks down into MPs through surface erosion (figure 1*a*), and how those MPs interact with MSAs to be exported to depth (figure 1*b*).

Large buoyant plastic items are not easily settled by passive sinking or biofouling [4,26] and their removal from surface water depends on their degradation into smaller particles that can be incorporated into MSAs (equation (1.1)) [24]. The model couples (i) a fragmentation scheme that redistributes eroded mass [25] into progressively smaller size bins (figure 1a), and (ii) a sedimentation scheme where MPs are small enough to be incorporated into and transported by MSAs [23]. Processes such as MP-MSAs aggregation, settling dynamics, MSA attenuation and MP detachment are explicitly represented in figure 1b.

### (a) Fragmentation model

This study assumes the degradation of plastic waste to produce small MPs predominantly occurs through UV radiation and following physical erosion/abrasion [27,28]. The rate at which MPs degrade within the model is set at 0.45% mass loss per month, following the experimental work of *in situ* degradation of PE at the ocean surface [29]. The abraded MPs are classified into five size bins from < 50 µm, 50−100 µm, 100−500 µm, 500−1000 µm, 1000−10 000 µm and the bin centres (25 µm, 75 µm, 300 µm, 1.25 mm and 6 mm). The bin centre is used to describe the MPs in each size bin, such as 75 µm MPs representing the MPs in the size bin of 50−100 µm. The fragmentation process was modelled using a negative binomial probability distribution, originally introduced by Charalambous *et al.* [30] and adopted in more recent plastic degradation studies [6,25]. This probabilistic approach captures the wide variability in observed environmental fragmentation patterns and ensures mass conservation by design.

The negative binomial fragmentation model is defined by two parameters:

— Fragmentation probability *p*, governing the likelihood of particles breaking into smaller fragments.
— Fragmentation index *f*, controlling the shape of the distribution and determining how concentrated fragmentation is towards smaller or intermediate bins.

The probability mass function (PMF) used to distribute fragments into size bin *k* is given by:


(2.1) $$PMF_{m}\left(k\right)=\;\frac{\Gamma \left(k+f\right)}{\Gamma \left(k+1\right)\Gamma \left(f\right)}p^{k}{\left(1-p\right)}^{f}.$$

This mass-based PMF is normalized to ensure that all degraded mass from a given bin is reassigned proportionally to smaller bins, preserving total mass. The size bins 25 µm and 75 µm are treated as terminal export states equation (1.1) and excluded from further degradation.

Since this study only tracks mass dynamics, we do not apply number-weighted scaling. Tracking of particle number would require such a transformation, but it is unnecessary for the purposes of mass redistribution and export modelling. This model captures gradual fragmentation and transfer of large buoyant plastics into smaller exportable MPs. All initial plastic mass is assumed to begin in the largest bin (6 mm), representing the release of a single 10 mm diameter PE particle.

### (b) Settling dynamics of small microplastics

Only two size bins of MPs (25 µm and 75 µm) are smaller than the size threshold (equation (1.1)) and can be incorporated into MSAs. These MPs are transported by MSAs according to a vertical sedimentation model (figure 1b). This model simulates vertical transport and settling dynamics of buoyant MPs through multiple cycles of incorporation and disaggregation with MSAs. MPs settle or rise based on their density relative to seawater [23,31].

The North Pacific Subtropical Gyre was selected due to its high surface plastic concentration [32,33]. The water column depth was set at 5100 m, with thermohalocline and density profiles obtained from the World Ocean Database (2020) [34]. Total particulate matter) concentration data was sourced from Subhas *et al.* [35], assuming a 1 mm diameter for MSAs due to their stability and prevalence [36]. The numerical concentration of MSAs (*N_MSA_*) was calculated: with an average TPM mass per aggregate (*N_MSA_ = TPM_con_/TPM_agg_*) of 2.5 µg [36].

Collision rates for MP incorporation into MSAs were computed from shear (*β_shear_*), differential settling (*β_settling_*) and Brownian motion (*β_Brownian_*) based on established flocculation theory [31,37,38]:


(2.2a) $$\beta_{shear}=1.3\;\gamma \;{\left(r_{MSAs}+\;r_{MPs}\right)}^{3},$$

(2.2b) $$\beta_{settling}=\frac{1}{2}\pi {r_{MSAs}}^{2}|v_{MSAs}-v_{MPs}|.$$

Shear rates (γ in s^−1^, gradient of velocity in the fluid) were depth-dependent, with a baseline of 0.2 s^−1^ [39,40]. The *β_Brownian_* is negligible and seven orders of magnitude lower than *β_shear_* and *β_settling_* (collector-limit) induced collision frequency. The shear rate distribution can be found in Wu *et al.* [23].

The settling velocity of particles be calculated based on Stokes’ law for aggregates/flocs [41,42]:


(2.3a) $$\begin{array}{r} v_{MSAs}=\frac{\left(\rho_{MSAs}-\;\rho_{sw}\right)\;{d_{MSAs}}^{2}g\;}{18\mu } \end{array},$$

(2.3b) $$\begin{array}{r} v_{MPs}=\frac{\left(\rho_{MPs}-\;\rho_{sw}\right)\;{d_{MPs}}^{2}g\;}{18\mu } \end{array}.$$

The $\rho_{MSAs}$, $\rho_{sw}$ and $\rho_{MPs}$ represent the density of MSAs, seawater and MPs, respectively—the density units are kg m^−3^; $d_{MSAs}$ and $d_{MPs}$ are the diameters (m) of marine snows and MPs, respectively; μ is the viscosity (kg m^−1^ s^−1^); g is gravitational acceleration (9.81 m s^−2)^. Once incorporated, the MPs settle with marine snows based on Stokes’ law (equations (2.3)). This model assumes that the marine snow settling velocity is not influenced by MPs in this scenario [21,43]. In this study, we set the effective density ($\rho_{MSAs}-\;\rho_{sw}$) at 1.2 kg m^−3^, and the resulting settling velocity of marine snow is also in the range of *in situ* measurement which is 1–368 m d^−1^ [36]. In this study, we only consider the fast-sinking marine snows as the only ones that can take buoyant MPs to the deep sea [44].

The attenuation ($L_{loss}$) is modelled as being directly linked to the diameter of the MSAs, assuming that fragmentation and settling-induced erosion is a first-order process proportional to the diameter rather than the volume when the MSAs settling below euphotic zone [9,45,46]:


(2.4) $$L_{loss}=\;Q_{10}^{\left(T-20\right)/10}R_{20}A\;,$$

where *R*_20_*A* = 0.1 (the unit is d^−1^) with the coefficient, *Q*_10_ = 2, which represents how much the attenuation increases by every 10°C increase in temperature [4,31,38], where *T* is the seawater temperature (^o^C) from [34].

When the MSAs are too small to hold the MPs, based on equation (1.1), the MPs become detached, regain buoyancy and rise in the ocean water, or the mass loss of marine snows is more than 40% of the initial value, and this is the end of the first settling event. After detachment, the MPs are released back into the water and start rising in the water column as the density of MPs is now lower than seawater equation (2.3). The detached buoyant MPs are available to be captured by ambient marine snows immediately at all depths based on the same collision functions equation (2.2). The re-incorporated MPs can regain the settling ability again.

Each settling cycle includes suspension of buoyant MPs, capture and settling with MSAs, MSA degradation and size reduction, and MP detachment (figure 1b). The second settling cycle begins with detached buoyant MPs rising again and the re-incorporation into marine snows (figure 1b). Each settling cycle results in MP settling for a certain depth and repeats until MPs hit the ocean bottom or reach the stable oscillation in the deep ocean. The long-term fate of MPs once they reach bottom of the ocean may be controlled by burial and resuspension [47–49], but this is not in the scope of this study.

### (c) Experimental set-up

The model runs for 100 yr to simulate the long-term fate of buoyant plastics in the ocean surface layer. Snapshots are taken at four time points: (i) 10 yr: approximately 30% of the parent plastic mass is lost; (ii) 30 yr, which represents the estimated half-life of floating plastic waste [8]; (iii) 75 yr, which reflects the lifespan of commercial plastic usage since the 1950s [50,51]; and (iv) 100 yr, which approximates a long-term equilibrium or deep-ocean accumulation. Degradation is updated monthly, while the sedimentation of incorporated MPs is resolved every 1 min to capture vertical movement with high temporal resolution. Once a particle reaches the ocean floor, it is assumed to be buried and is no longer tracked (figure 1b).

### (d) Sensitivity analysis

To evaluate the robustness of the model outputs, we performed a sensitivity analysis by varying three key parameters of the fragmentation model: the degradation rate, the fragmentation probability *p* and the fragmentation index *f.* (i) Degradation rate was tested across three values: 0.00045, 0.0045 (default) and 0.045 month^−1^, corresponding to low, medium and high erosion scenarios informed by empirical studies [7]. (ii) *Fragmentation probability p* influences how likely the degraded mass fragments into smaller pieces. We explored values of 0.1, 0.3 and 0.5, representing weak and strong fragmentation tendencies. (iii) *Fragmentation index f* controls the shape of the redistribution profile in the negative binomial distribution. Lower values (e.g. 0.1) favour small particle formation, while medium and higher values (e.g. 0.3 and 0.5) spread mass more evenly across bins.

## 3. Results

### (a) The fragmentation of large plastics

**Figure 2.**
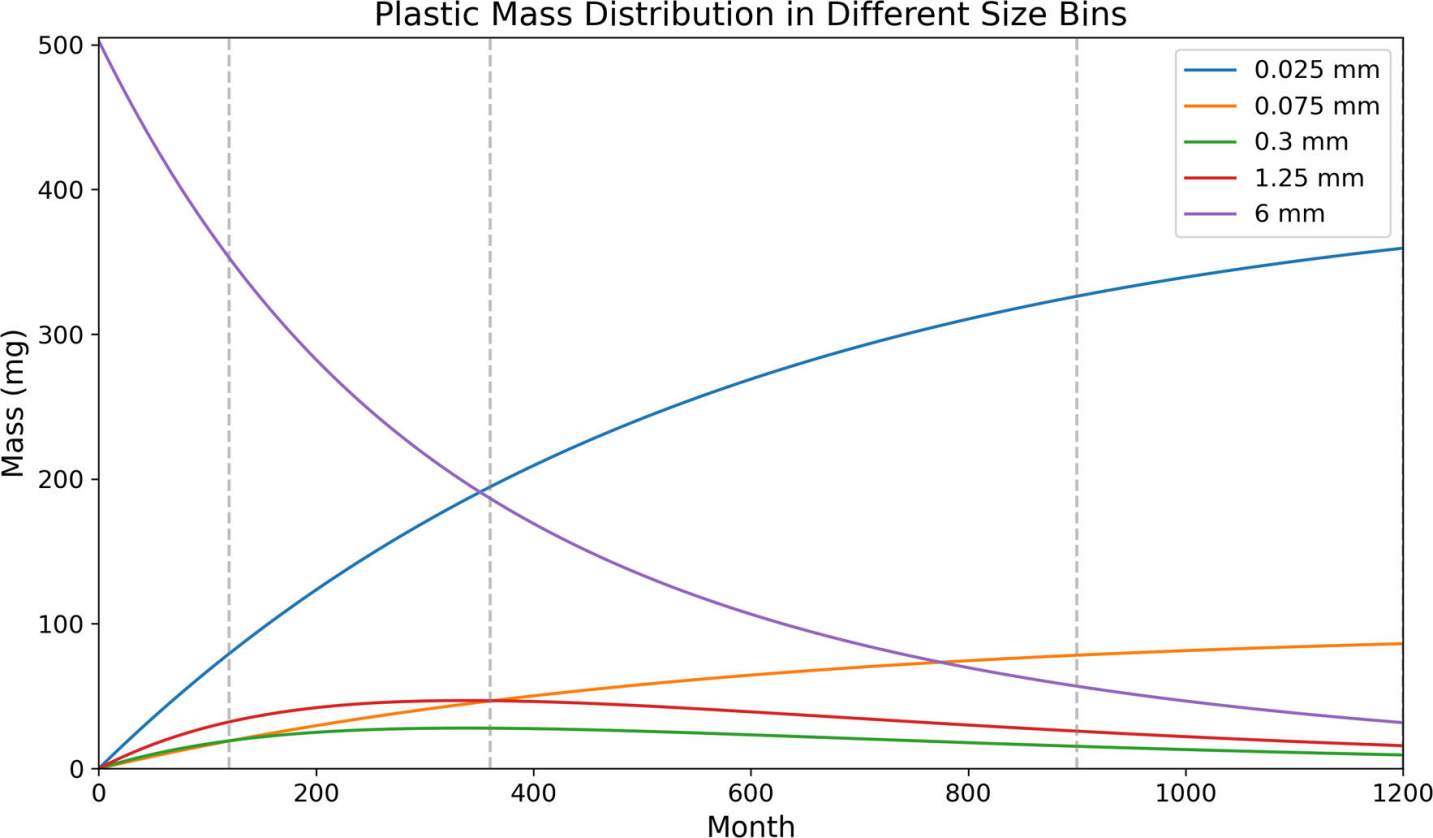
The size distribution of fragmented MPs in different size bins with time for 100 yr. The initial size of parent plastic is 10 mm with a mass of 502.64 mg.

After 10 yr of degradation the mass of the parent plastic particle has decreased from 502.64 mg to 353.80 mg, with a mass loss of approximately 30% (figure 2). Small MPs that can be incorporated into MSAs and settle out of the ocean surface, are made of 19.4% of total plastic mass based on equation (1.1). The remaining lost mass is distributed among the other size bins, with 78.79 mg, 18.91 mg, 19.09 mg and 32.08 mg placed within the 25 µm, 75 µm, 300 µm and 1.25 mm bins, respectively (figure 2). After 30 yr, the mass of the parent particle has decreased to 187.11 mg, which represents a loss of 62.7% of the original mass. The proportion of small MPs increases to 47.9% according to equation (1.1). After 75 yr the parent particle has a mass of 56.99 mg, accounting for 11.3% of original mass, and the numbers in 0.3 mm and 1.25 mm bins are 12.39 mg and 25.88 mg, respectively. The majority of buoyant MPs is degraded into the smallest size ranges, accounting for 80.5% of the original mass being able to be removed by MSAs. Running the model for a century, only 31.76 mg of the parent particle remains, accounting for a mass loss of 93.7%. The majority of these degraded MPs now fall into the smallest particle size bins, and across the whole system 359.40 mg are in the 25 µm bin, and 86.26 mg are in the 75 µm bin, which are subsequently removed from ocean surface by MSAs.

Throughout the model run, the mass of the smallest sizes of MPs (25 µm and 75 µm) increases consistently because the larger plastic groups keep releasing smaller particles in a nonlinear process. The mass of mid-sized MPs (0.3 mm and 1.25 mm) is increased initially because of the abrasion of the largest plastic particles. Subsequently, the mass of these two size bins decreases because of the continued degradation of particles to ever smaller sizes (figure 2).

### (b) Fate and transport of small and large MPs

**Figure 3.**
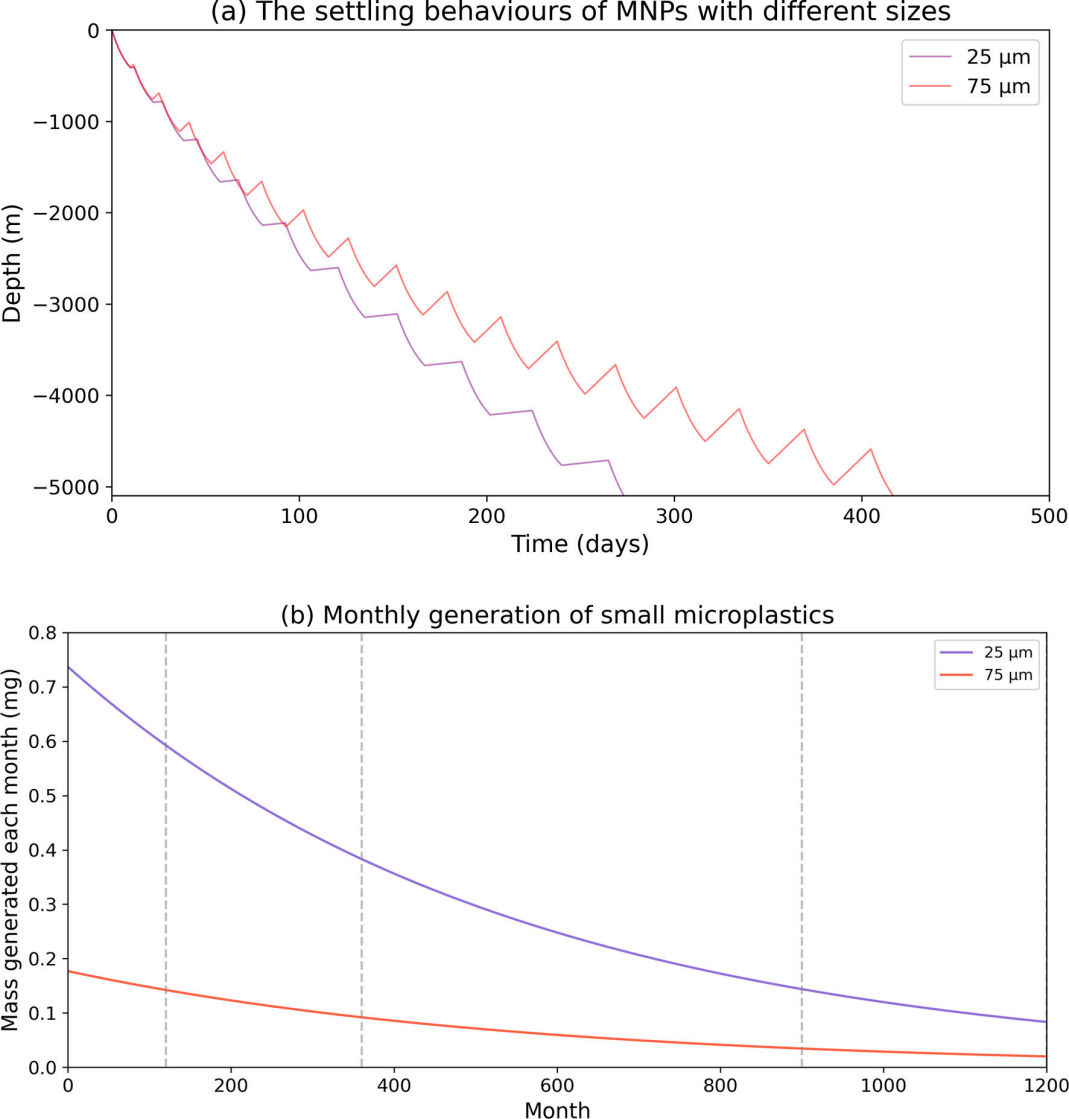
(a) The one-dimensional trajectories of 25 µm (purple) and 75 µm (red) MPs from ocean surface to deep-sea sediment (depth is 5100 m). (b) The mass of 25 µm MPs and 75 µm MPs deposited into the ocean by interaction with MSAs.

MP fragments of 25 µm and 75 µm bins can be incorporated into MSA and then settle out of the ocean surface. The tracks of small MPs were plotted based on the depth of MPs and time (figure 3a). The sedimentation model shows that the small MPs can be taken to the deep-sea sediments by multiple settling cycles. This takes approximately 80 d for 25 µm MPs to get to a sea depth of 3000 m and 289.4 d to reach the bottom of the ocean. The exported small MPs from the ocean surface via MSAs settling decreases over time. In the first month, the exported 25 µm MP mass is 0.74 mg, while this value decreases to 0.59 mg after 10 yr of simulation (figure 3b) which is significantly lower than the first month. The exported masses are 0.14 mg and 0.08 mg after 75 yr and 100 yr of fragmentation, respectively (figure 3b).

The 75 µm MPs take approximately 90 d to reach the deep sea (more than 2000 m) and 416.7 d to reach the ocean bottom (figure 3a). The trend for mass export to the ocean surface via MSAs settling is similar to that of 25 µm MPs. The exported mass of the first month is 0.18 mg, while this value is decreased to 0.14 mg after 10 yr of fragmentation. The values are 0.03 mg and 0.02 mg after 75 yr and 100 yr of simulation (figure 3b). The mass distribution to small MP bins decreases with time as the parent particle mass decreases with time, and the supply by the fragmentation is decreased (figure 2). Even after 100 yr of fragmentation, the parent particle still exports small MPs to the ocean subsurface, suggesting the persistent influence of the plastic waste to the ocean ecosystem on these ‘century-long’ time scales.

### (c) Distribution of plastic mass

**Figure 4.**
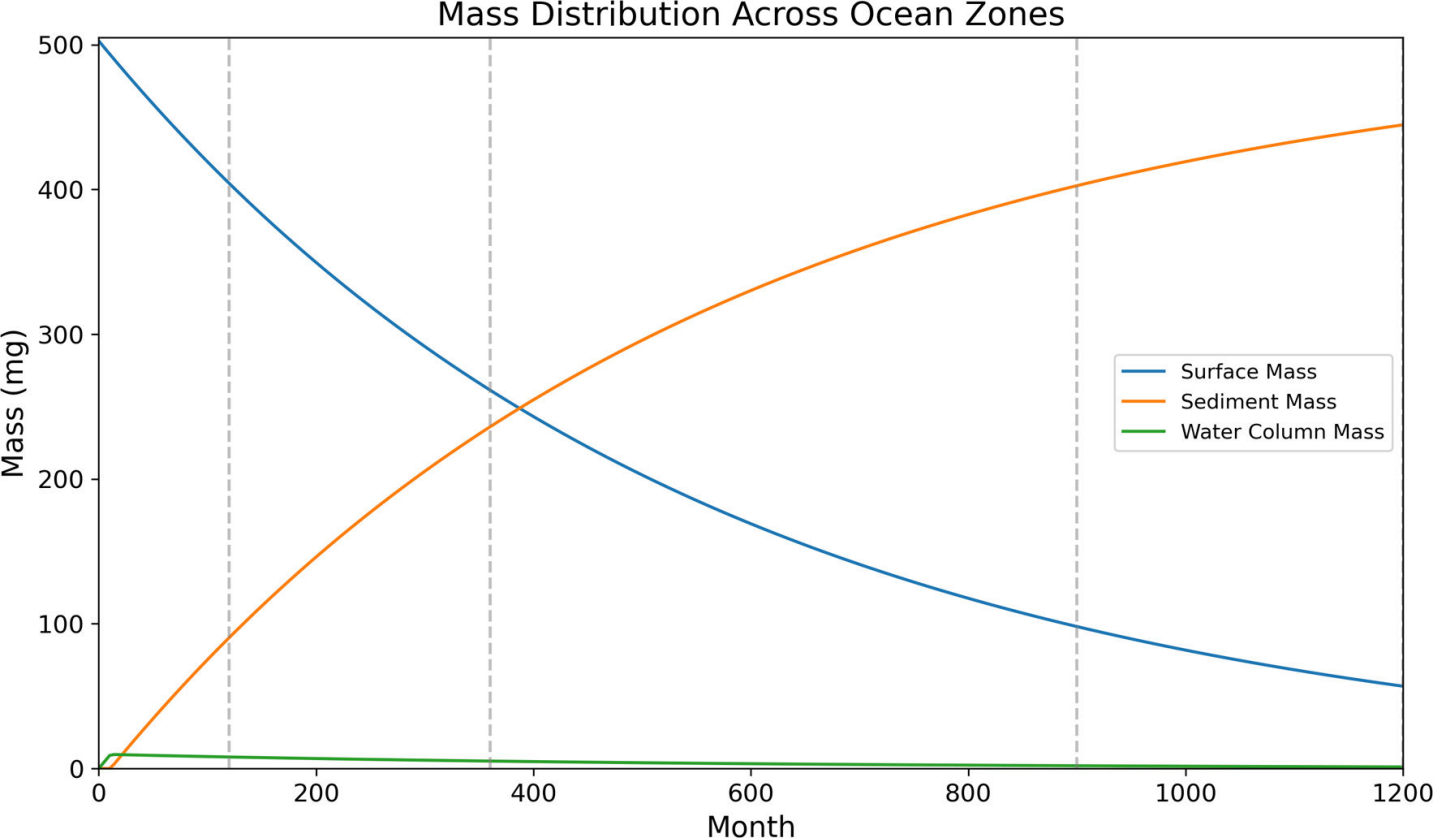
Temporal evolution of plastic mass distribution across ocean surface, water column and deep-sea sediment over 100 yr. The model tracks the fate of a 10 mm PE particle as it fragments into MPs and redistributes vertically. Surface mass (blue) decreases gradually as fragmentation occurs, while sediment mass (orange) increases due to the incorporation of small MPs into MSAs and subsequent settling. Water column mass (green) remains low throughout, reflecting rapid vertical transfer of exportable MPs. Dashed vertical lines mark 10, 30, 75 and 100 yr for reference.

The mass of MPs at the ocean surface keeps decreasing because the parent plastic mass is fragmenting into small MPs, which are subsequently removed by marine snow. The degradation and incorporation processes are very slow based on our simulations, and it takes a decade to remove only 19.4% of the plastic mass from the ocean surface, and after 100 yr, 57.00 mg of plastic residue remains at the ocean surface (figure 4). This means that if all plastic pollution input into the ocean were stopped, there would be a lag time of more than a century before extant plastics all reached the ocean subsurface and bottom sediments (figure 4). The MPs that can be incorporated are transported to the subsurface by the sedimentation of MSAs, and the mass of small MPs exported into the ocean subsurface increases with time. The total mass of MPs reaching the ocean subsurface begins at 0 mg in the first month and reaches 444.53 mg (88.4% of the total mass) after 100 yr of simulation (figure 4).

When MPs reach the bottom of the ocean, the sediment starts accumulating MP mass. The mass in the sediment follows the opposite trend as plastic mass at the surface, but with a lag of 10 months for 25 µm MPs and 14 months for 75 µm MPs due to differences in settling rate by mass (figures 3a and 4). The masses of small MPs reaching the sediment increases, from 90.44, 236.05, 402.64 and 444.53 mg after 10, 30, 75 and 100 yr of simulation, respectively.

The model can also be used to explore the mass of MPs within the water column at a given time step. Two size categories of MPs (25 µm and 75 µm) are observed to be travelling in the ocean water column. Initially, the mass of these two size fractions increases rapidly, reaching a peak after 11 months (25 µm) and 15 months (75 µm) at 9.69 mg for whole water column accounting for 1.9% of the total mass. After this point, the MP mass in the water column decreases, in line with the steady reduction in input mass at the surface over the duration of the model run. After 100 yr of simulation, the ratio of mass between the surface and deep ocean MPs and that settling within the water column decreases to 0.2%, which demonstrates that the majority of buoyant plastics are either on the ocean surface or entering deep ocean sediments, with a negligible proportion stored in the water column based on our parameterizations.

### (d) Sensitivity analysis

The model outcomes are highly sensitive to changes in degradation and fragmentation parameters. Varying the degradation rate over two orders of magnitude revealed it to be the dominant control on surface plastic longevity. With a low degradation rate (0.00045 month^−1^), 404.46 mg of the original plastic remained at the surface after 100 yr. At the default rate (0.0045 month^−1^), this dropped to 56.99 mg. Under a high degradation scenario (0.045 month^−1^), the surface plastic mass was nearly eliminated (less than 0.0001 mg), indicating almost complete fragmentation and export. The fragmentation probability also significantly influenced outcomes. A lower probability (*p* = 0.1) led to accelerated mass loss, with only 3.09 mg remaining at the surface. By contrast, a higher value (*p* = 0.5) retained 272.90 mg, as more mass was redistributed into mid-sized bins that remain buoyant for longer. Changes in the fragmentation index had a moderate effect. Lower values (*f* = 0.1) favoured formation of smaller MPs, resulting in more rapid surface mass loss (56.99 mg remaining). Higher values (*f* = 0.3, 0.5) preserved more mass at the surface (up to 257.30 mg) by spreading redistribution across a wider size range. Together, these results demonstrate that degradation rate exerts the strongest control on plastic persistence at the surface, with fragmentation parameters shaping the pace and pathway of size reduction and export.

## 4. Discussion

**Figure 5.**
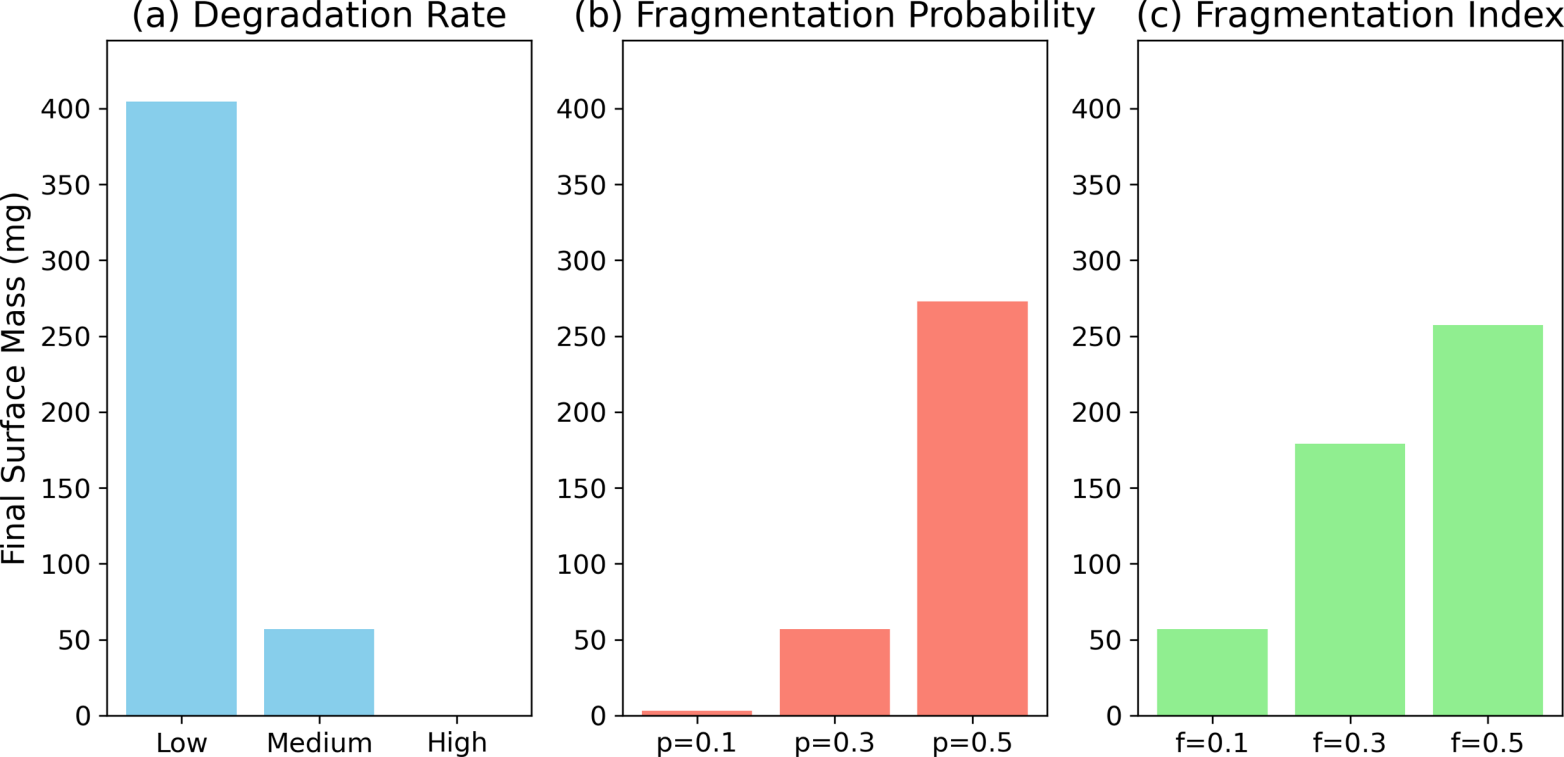
Sensitivity of final surface plastic mass to key fragmentation parameters. (a) Increasing degradation rate accelerates fragmentation into small MPs, sharply reducing the mass retained in surface-associated bins after 100 yr. (b) A higher fragmentation probability *p* concentrates redistribution into mid-sized particles, slowing export and resulting in greater surface retention. (c) A higher fragmentation index *f* spreads redistributed mass more evenly across size bins, preserving larger fractions of surface-retained plastic. Final values represent the remaining mass in the 0.3 mm, 1.25 mm and 6 mm bins after 100 yr. Results are reported as the remaining mass in surface-representing bins (0.3 mm, 1.25 mm, 6 mm) after 100 yr.

This study presents the first mechanistic model that couples large plastic degradation and fragmentation with a size-selective sedimentation framework, enabling us to predict the long-term fate of buoyant plastic at the ocean surface. Previous studies used biofouling to explain how these buoyant large plastics reach the ocean subsurface [4,38,52], while recent studies note that only very small MPs can be transported by this process [5,23,24], and no mechanism to explain the transport of MPs to deep ocean sediments through biofouling has been offered [1,26,38]. According to published results, small MPs are dominant at the ocean bottom [15], and large buoyant plastics rarely reach the deep ocean [13,14]. The plastic waste at the ocean surface is gradually abraded and degraded into the smaller MPs, and then the theoretical settling model is applied to explain how they reach the deep ocean (figures 3a and 4). The modelling output is consistent with previous environmental monitoring results that the sediments have extremely high concentrations of small MPs when compared with the water column [12,20,53,54]. In this study, we found the buoyant plastic mass in the water column accounts for less than 2% (figure 4), and this is consistent with previous results that the sediment acts as an MP sink and accumulates most of the MPs [13–15]. The settling of incorporated MPs from the ocean surface to sediment takes approximately 1 yr for 25 µm and 75 µm MPs (figure 3a), and this is one order of magnitude less than the time required for large plastics to fragment and reach size thresholds at which they can be readily incorporated into MSAs, and hence begin vertical settling (figure 2). After 10 yr of fragmentation, the parent plastic microsphere has only lost 30% of its mass (figure 3). In addition, the fragmentation curve has a similar pattern to the mass distribution curve in the sediments (figure 4). This means that degradation is the dominant process contributing to the fate and transport of plastic wastes in the ocean which agrees with our sensitivity analysis (figure 5). Therefore, the key to removing plastic from the ocean surface is determined by degradation time scales. The distribution of MPs in the subsurface, surface and sediment is similar to the fragmentation curves, which means that the settling of the MPs only slightly influences the mass distribution in the water and sediment, while the fragmentation time scale is the key to determining the fate of MPs according to our theoretical model. Note that the scenario in the real ocean with different ocean biogeochemical parameters and three-dimensional transport is very complex, but still governed by the fundamental processes represented in our model.

The parent particle still exports small MPs into the subsurface after 100 yr of degradation. This suggests the natural removal of buoyant plastic wastes at the ocean surface is a long-term process, and slow degradation is the only natural way to remove the persistent polymers from the ocean surface [52,55]. Therefore, even if society stops all the plastic input into the ocean immediately, the environmental consequences of plastic waste will persist for over 100 yr. However, currently, the rates at which plastic waste is released to the global ocean are increasing [1,33,50], demonstrating the scale of the environmental challenge that plastic pollution poses. However, the rate of increase in MP concentrations is expected to outpace any corresponding change in MSAs, and the ratio of MPs to MSAs is likely to rise over time, potentially straining the capacity of the biological pump in oligotrophic regions such as ocean gyres. Therefore, if this rate of release continues to increase, a threshold will eventually be crossed, where the concentration of small MPs in the water column will outstrip the rate of incorporation and settling by MSAs [11,22]. This future scenario of an excess of MPs relative to MSAs will slow down their settling rates and hence reduce the carbon export and disturb the ocean biogeochemical cycles. Therefore, the key is reducing plastic production and better managing plastic waste, which stops plastic pollution before it can reach the marine environment and begin to degrade.

A future aim of the discipline should be to couple this model with a three-dimensional general ocean circulation model to better predict the degradation and settling of buoyant MPs. Such a model would allow the prediction of MP distributions within the world oceans, supporting targeted clean-up operations and identifying pollution hotspots. The validation of these predictions will require more sampling of MP concentrations with depth across a range of ocean settings. The one-dimensional model presented here represents a crucial step towards these aims, although additional complexity is required to take these next steps. The current model represents the fate of a single particle, representing one polymer and one degradation process; by expanding future models to represent multiple polymer types and morphologies of MPs and macroplastics, clear priorities for waste reduction policies can be implemented, targeting the waste with the longest residence time on the ocean surface.

## 5. Conclusions

We present a coupled degradation and sedimentation model to explain how buoyant and large plastic wastes are removed from the ocean surface, filling a critical gap in our understanding of plastic transport, supported by previous experimental data and field observations from a variety of ocean settings. The longevity of plastic waste at the ocean surface is limited by the degradation rate of plastics. Plastics are observed to accumulate at the ocean surface, but our model demonstrates that these plastic wastes gradually transfer into the deep ocean and sediments via a process of degradation, multiple cycles of aggregation–disaggregation with MSA and sedimentation. These patterns correspond to the observations of MP concentrations throughout the water column that suggest that less than 2% of MPs by mass are in suspension. The model presented here demonstrates that after 100 yr of simulation, assuming no further inputs of plastic pollution to the ocean, a proportion of the initial 10 mm PE plastic microsphere persists on the ocean surface, exporting small MPs into the ocean subsurface and posing risks to marine ecosystems. These results outline the severe environmental effect that plastic pollution is having on marine ecosystems and show that it will continue long after initial waste release is halted.

## Data Availability

All simulation codes used in this study are openly available via Zenodo at the following [56].
